# COVID-19 and Pulmonary Tuberculosis Coinfection in a Moroccan Patient with Pulmonary Embolism: A Case Report and Literature Review

**DOI:** 10.1155/2022/1522876

**Published:** 2022-07-30

**Authors:** Imane Zouaki, Zakaria Chahbi, Mohamed Raiteb, Mohamed Zyani

**Affiliations:** Department of Internal Medicine, Avicenne Military Hospital, Marrakech, Morocco

## Abstract

Emerging cases of coinfection of coronavirus disease 2019 (COVID-19) and tuberculosis (TB), although rare, have attracted the attention of health systems around the world and have arisen many concerns about the diagnosis, treatment, and prognosis of this coinfection especially in high TB burden countries. Here, we report a rare case and, to the best of our knowledge, the first reported case in Morocco of simultaneous diagnosis of an active pulmonary TB infection and a COVID-19 pneumonia. We present a case of a sixty-seven-year-old male patient who was admitted to our COVID-19 emergency department with a diagnosis of COVID-19 pneumonia, confirmed by nasopharyngeal swab's polymerase chain reaction (PCR) for detection of SARS-CoV-2. The atypical radiological findings suggested a TB coinfection which was later confirmed by sputum cultures and Xpert MTB/Rif assay. The patient also presented some complications including thrombosis of the left leg, pulmonary embolism and inaugural ketosis. Treatment was administered as per local protocols: broad spectrum antibiotics, corticosteroids, fixed dose-combination of antituberculosis treatment along with hydration and insulin therapy for ketosis treatment and anticoagulation. The patient was discharged after twenty-three days of hospitalization. Due to the currently limited data, further studies are necessary to establish any possible correlation between COVID-19 infection and the progression of a latent and/or the severity of an active TB infection.

## 1. Introduction

The coronavirus disease 2019 (COVID-19) caused by the novel severe acute respiratory syndrome coronavirus 2 (SARS-CoV-2) is still spreading across the world since its start on December 2019 in Wuhan city, China, and then declared a pandemic by the WHO on March 2020 [1]. The clinical features are dominated by respiratory and flu-like symptoms such as fever, cough, and fatigue. Meanwhile, tuberculosis is still a global burden affecting millions of people every year with the highest mortality rate of any infectious disease [2]. The concurrence of these two pandemics has arisen many concerns in terms of clinical management, differential diagnosis, treatment, and prognosis, especially in high TB burden countries [3]. There is a scarcity in the current literature concerning cases of coinfection of COVID-19 and TB. Here, we report a rare case of simultaneous diagnosis of an active pulmonary TB infection and COVID-19 pneumonia with thrombotic complications.

## 2. Case Presentation

On December 30^th^, 2020, a sixty-seven-year-old Caucasian male, with no medical or surgical history, presented to our emergency COVID-19 department with persistent dyspnea at rest, along with chest pain, and a persistent cough initially dry becoming productive fifteen days before admission, showing no improvement on clavulanic acid-amoxicillin taken by the patient on self-medication, in a context of fever and night sweats, and deterioration of general status including a weight loss of 10 kg, fatigue, and anorexia. However, the patient had not shown any signs of otorhinopharyngeal viral infection (no anosmia, no taste alteration, no sore throat, and no rhinitis) and had not reported any known close contact with a COVID-19 except that his son had been treated for pulmonary TB infection a year earlier.

Upon his admission, physical examination revealed a conscient bedridden patient, febrile at 38°C, tachypneic at 30 cpm, saturation levels at 86% on ambient air, tachycardic at 100 bpm, and normotensive with bilateral rhonchi, a swollen painful left leg with positive Homans' sign and vesicular eruption on his abdomen. On another note, his admission's capillary blood glucose was as high as 4 g/l and his urine strip revealed 2 positives for ketones and 2 positives for glucose, thus revealing an inaugural diabetic ketosis. His EKG and cardiac enzymes were normal.

**Figure 1:**
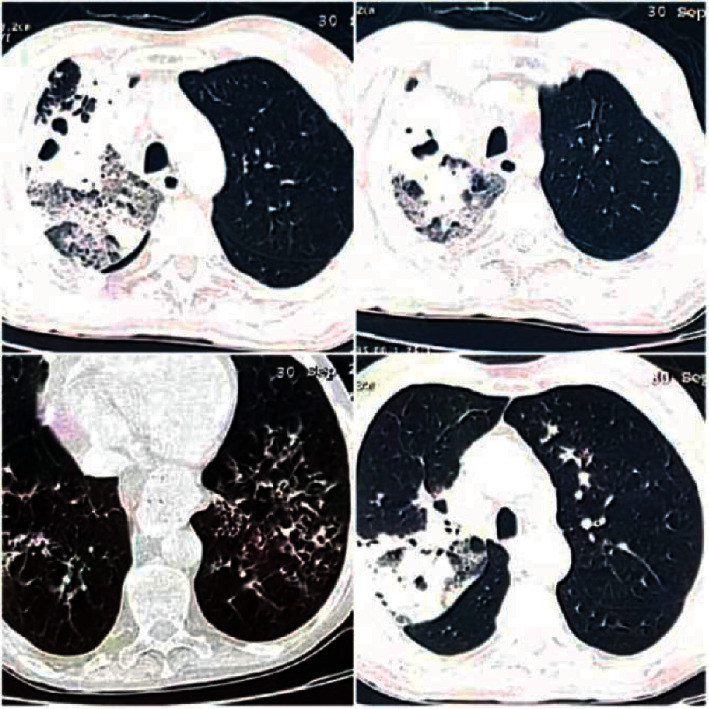
CT scan images showing foci of pulmonary condensation and cavitations of the right upper lobe with diffused ground-glass opacities and multiple bilateral foci of nodules and micronodules suggesting CO-RADS 5 with signs of active tuberculosis.

His laboratory findings showed an elevated white blood cells count (10240/ml) (normal: 7000–10000/ml) with high neutrophilic count, a microcytic hypochromic anemia at 10.7 (normal: 13–18 mg/dl), lymphocytopenia at 710 (normal: 1500–4000/mL), an elevated C-reactive protein at 208 (normal:<5 mg/l), normal procalcitonin level 0.26 (normal <5), elevated lactate dehydrogenase at 371 (normal: 135–225 U/L), elevated ferritin at 764 ng/ml (normal: 30–400 ng/ml), blood sugar at 2.29 g/l, and bicarbonates at 22.75 (normal: 22–30). His renal and liver full workup was normal. HIV and other viral serologies also came back negative. His chest computed tomography (CT) scan showed foci of pulmonary consolidation with diffused ground-glass opacities and multiple bilateral nodules and micronodules all in favor of a CO-RADS 5 with signs of active tuberculosis (Figure 1).

His nasopharyngeal swab for COVID-19 reverse transcriptase-polymerase chain reaction (RT-PCR) came back positive, thus confirming a COVID-19 infection. Also, his sputum for MTB PCR was positive with no rifampicin resistance detected revealing a concomitant infection with *Mycobacterium tuberculosis*. A venous doppler ultrasound of his left leg revealed a total thrombosis of the superficial femoral, popliteal, and sural veins.

The patient was admitted to one of our COVID-19 isolation units and was put under oxygenotherapy via a high flow nasal cannula at 6 liters/min. He was started on broad spectrum antibiotics (2 g of ceftriaxone) for 10 days, corticosteroids (methylprednisolone) 80 mg for 5 days, 40 mg of prednisolone orally, a fixed dose-combination of antituberculosis treatment (isoniazid + rifampicin + pyrazinamide + ethambutol), and anticoagulation with low-molecular weight heparin enoxaparin at 0.6 IU^*∗*^2 per day and was prescribed support stockings. Intravenous hydration was started as per ketosis correction protocol with correction of hydroelectrolytic disorders and intravenous and then subcutaneous insulin therapy with the following regimen: basal insulin in the evening with boluses of rapid insulin three times a day, depending on the patient's capillary blood sugar.

**Figure 2:**
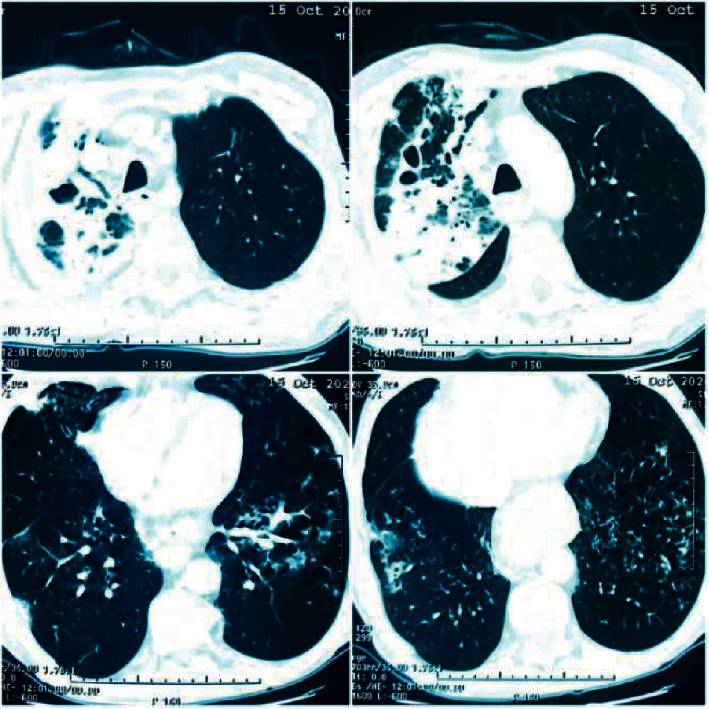
CT scan images showing bilateral micronodules and bronchial dilation in the right upper lobe with parenchymatous opacification. Endoluminal defects of the left pulmonary base suggesting a pulmonary embolism with signs of active TB infection.

The evolution was favorable with apyrexia on the second day of treatment, the oxygenotherapy was discontinued progressively over 10 days, the patient's COVID-19 PCR came back negative on day 15th of his admission, and his chest CT scan showed endoluminal defects of the segmental arteries of the left pulmonary base, focal cystic dilatation of the upper right lobe with focal parenchymatous consolidation, multiples bilateral micronodules, and lymphadenopathy in Barety's compartment all in favor of a pulmonary embolism with signs of active tuberculosis (Figure 2).

After a stay of 23 days, the patient was discharged with a prescription of anti-TB therapy, antivitamin K (started on day 15th of admission), and oral corticosteroids (prednisone) for 10 days with progressive degression and a basal bolus insulin regimen.

## 3. Discussion

Our patient presented a rare case of simultaneously diagnosed coinfection of COVID-19 and active pulmonary TB. This coinfection was described in both genders and in all age groups including a three-month-old Gambian patient, with a slight predominance in males and migrants [4]. These cases were only reported in some countries like Italy [5–9], Singapore [10, 11], India [12–15], China [16–19], Brazil [20, 21], Turkey [22, 23], Saudi Arabia [24–26], and Mexico [27].

In Table 1, we describe clinical features and radiological findings as well as treatment options and outcome in our literature review of reported cases of this coinfection. The most common symptoms of COVID-19 include fever, cough, and dyspnea [56]. These symptoms are also common in TB infection [57] which makes TB diagnosis rather difficult and sometimes even delayed as in some cases. Our patient as well as multiple reported cases have presented unusual symptoms including night sweats, anorexia, weight loss, and hemoptysis (Table 1). These clinical features are typical of tuberculosis [57] and thus are important to look for when a patient presents respiratory complaints especially in high TB burden countries like ours.

In our case, the diagnosis of COVID-19 was confirmed by RT-PCR for SARS-CoV-2 done upon admission, systematically as per national protocols due to the ongoing pandemic. The complementary CT scan showed pulmonary condensation and diffused ground-glass opacities, commonly described in COVID-19 pneumonia [58]. It also revealed bilateral nodules and micronodules which are characteristic of an underlying active TB infection [59]. In the reported cases, other radiological findings suggesting TB infection included infiltrates, consolidation, cavitation, pleural effusion, miliary, and calcification (Table 1). These previous aspects are atypical of COVID-19 and their presence signs a TB coinfection [59] in need of further confirmatory tests.

The TB diagnosis in our case was first brought up by the atypical CT scan findings and was later confirmed by GeneXpert MTB/RIF assay and sputum cultures coming back positive for drug susceptible M.tb (*Mycobacterium tuberculosis*) like most of the reported cases (Table 1). However, drug resistance was reported in some cases (Table 1).

In our case, we noted lymphocytopenia, neutrophilia, increased inflammatory markers (CRP and ferritinemia), and LDH which was described in COVID-19 infection and linked to severe disease and higher mortality [60]. On the same note, an elevated D-dimers level was also related to worse prognosis and to high probability of coagulopathy [60]; although not measured in our case, they would have been elevated since our patient had already presented thrombotic complications.

Although thrombotic complications are frequent in COVID-19 infection, their exact incidence has not yet been determined. However, it is noted that their rate is higher in critically ill COVID-19 patients admitted to ICU wards in comparison to non-ICU patients [61]. Their pathogenesis could mainly be related to local factors including inflammation and immunothrombosis induced by the viral infection and/or to the usual thrombotic factors such as prolonged bed rest, age, and certain comorbidities [61, 62]. Our patient has presented PE complicating DVT, but studies show that PE could occur independently from DVT which supports the hypothesis of in situ thrombosis mechanism [61, 62]. Fortunately, our patient has made an uneventful recovery, but these complications and specifically PE are considered independent factors of morbidity and mortality in COVID-19 patients [63], highlighting the importance of the close monitoring and the development of prophylactic anticoagulation protocols especially for critically ill patients.

There is still no consensus considering the treatment of COVID-19 and tuberculosis coinfection. Our patient received the classic quadruple antituberculosis regimen (isoniazid + rifampicin + pyrazinamide + ethambutol) along with broad spectrum antibiotics for the COVID-19 pneumonia as per the national protocol in Morocco. In the cohort of Tadolini et al., while the antitubercular treatment was the same for drug susceptible M.tb, the reported treatment for COVID-19 was of different combinations of hydroxychloroquine, azithromycin, and lopinavir/ritonavir: 17 patients received a monotherapy, 9 a combination of two drugs, and 2 received 3 or more drugs [28]. Antiretroviral therapy and hydroxychloroquine are empirically used for COVID-19 treatment; however, they were shown to have interaction with antitubercular drugs, especially rifampicin, isoniazid, and second-line treatments [64]. Since these drug interactions have not been fully elucidated, their use in our case was avoided.

The use of corticosteroids in COVID-19 is recommended in certain cases to modulate the inflammatory process as in our national COVID-19 protocol. Many cases in literature suggest the possibility of TB reactivation under these immunosuppressive treatments [65], but due to limited data, no conclusion can be drawn about this subject. Recommendations are to administer corticosteroids in a low dose and for a short period of time when it is indicated to avoid inducing immunosuppression and risk opportunistic infections such as TB [66].

In the study by Tadolini et al., 9 patients had both diseases diagnosed within the same week including 4 on the same day, and 14 had COVID-19 first [28]. However, the authors failed to show the role of COVID-19 in the progression of TB due to the study limitations. The relationship between COVID-19 and the onset of TB infection is still debatable, but it seems to be bidirectional. Since both infections depend on cellular immunity, temporary immunosuppression caused by SARS-CoV-2 increases susceptibility for new TB infections or reactivation of latent TB infections and vice versa [34].

In the same cohort, the mortality rate was as high as 6/49 (12.3%) and was reported in aging patient with 1 or plus comorbidities [28]. The risk factors for this coinfection are comparable to those of TB without COVID-19 including age, COPD, HIV, smoking, diabetes, hypertension, and renal failure. Our patient checked two of these factors, those of age and diabetes, and fortunately presented a moderate pneumonia that did not require intensive care with a favorable evolution.

In conclusion, the possible overlap of symptoms of COVID-19 and TB must alert clinicians to think of TB coinfection when confronted with atypical clinical or radiological presentations, especially in migrants and high TB burden countries. Larger studies are needed to fully to explore and establish clear treatment and prevention protocols for this coinfection.

Written informed consent was obtained from the patient for using images and other relevant data for publication in this study.

## 4. Conclusion

In the mist of the ongoing pandemic, as all resources are being allocated to the fight against COVID-19, the emerging cases of COVID-19 and TB coinfection is ought to remind us of an evenly important fight, that against TB. Therefore, we strongly recommend testing for both diseases when faced with respiratory symptoms especially in high TB burden countries like ours or at least before atypical clinical or radiological presentations. The available data about these cases is limited still, and further studies are necessary to better comprehend the effects of COVID-19 on TB and vice versa.

## Figures and Tables

**Table 1:** Information on the reported cases.

Study	Country	Number of patients	Median age	Comorbidities	Clinical presentation	Radiological findings	Site of TB	COVID treatment	TB treatment	Mortality
Tadolini et al. [28]	Multicenter	49	49	8 COPD/asthma, 8 diabetes, 6 HIV, 5 kidney disease, 7 liver disease, 10 alcoholism, 20 smoking, 4 drug abuse	32 fever, 27 cough, 17 dyspnea	21 bilateral GGO, 23 cavitation, 25 infiltrates	36 pulmonary, 1 extrapulmonary, 12 both	22 HCQ, 12 antiviral therapy, 7 AZT, 1 other	NM	6 deaths
Gupta et al. [12]	India	22	44	3 diabetes, 4 hypertension, 2 seizure disorder, 1 hypothyroidism	22 fever, 11 cough, 7 dyspnea, 1 weight loss, 1 headache	14 bilateral infiltrates typical of COVID, 9 pulmonary fibrosis, 3 cavitations, 6 infiltrates/consolidation, 2 pleural effusion	17 pulmonary, 4 extrapulmonary; 1 disseminated	NM	21 (RIF, INH, PYR, ETB), 1 MDR therapy	6 deaths
Stochino et al. [5]	Italy	20	40	1 COPD, 4 diabetes, 1 psoriasis, 1 hypertension, 1 sickle cell disease	12 fever, 9 cough, 3 dyspnea, 3 chest pain, 2 headache, 1 conjunctivitis, 3 asymptomatic	1 GGO, 3 interstitial syndrome, 8 cavitations, 14 nodules, 1 pleural effusion, 1 calcification, 2 miliary	16 pulmonary, 1 extrapulmonary, 2 both, 1 disseminated	20 HCQ	14 (RIF, INH, PYR, ETB), 2 MDR therapy, 4 tailored therapy	1 death
Gül et al. [22]	Turkey	16	40.68	2 smoking, 2 asthma, 2 cardiac disease, 2 diabetes	3 fever, 7 cough, 3 dyspnea, 2 headache, 4 anorexia, 1 hemoptysis, 1 skin rash, 4 asymptomatic	9 GGO, 6 infiltration, 4 cavitations, 1 nodules, 1 pleural effusion	9 pulmonary, 5 extrapulmonary, 1 both	10 HCQ, 8 FAV	13 (RIF, INH, PYR, ETB), 1 (RIF, INH, PYR, STR), 1 (RIF, INH, STR, ETB)	1 death
He et al. [16]	China	3	56.3	2 smoking	3 fever, 3 cough, 1 chest pain, 2 dyspnea	3 GGO, 1 cavitation	3 pulmonary	3 LOP/RIT, Arbidol	1 (RIF, INH, PYR, ETB)	0
Liu et al. [17]	China	3	40	1 MDR-TB, 1 *Aspergillus* infection	3 fever, 2 dyspnea, 2 cough, 1 myalgia, 1 sore throat, 1 chest pain, 2 respiratory distress, 2 hemoptysis	3 GGO	3 pulmonary	1 AZT, 3 Arbidol, 3 MOX, 2 LNZ	1 (ETB/PYR/AMK/LEV), 1 CS, 1 CFZ, 1 LNZ	0
Yao et al. [18]	China	3	50.3	2 smoking, 1 diabetes	3 fever, 3 cough, 3 weight loss, 1 night sweat	3 GGO, 2 pleural effusion	3 pulmonary	2 LOP/RIT, 1 UMF HCL, 2 IFN-*α*	2 RIF, INH, PYR, ETB	1 death
Cao et al. [19]	China	1	47	Asthma	Unwellness, poor appetite	Cavern calcification	Pulmonary	LOP/RIT	RIF, INH, PYR, ETB	0
Tham et al. [10]	Singapore	4	31.75	NM	4 fever, 4 cough, 1 dyspnea, 1 weight loss	1 GGO, 1 cavitation, 2 pleural effusion,3 consolidations	4 pulmonary	NM	4 RIF, INH, PYR, ETB	0
Al Lawat et al. [29]	Oman	2	49	1 smoking, 1 diabetes, 1 hypertension	2 fever, 2 cough,1 dyspnea, 1 chest pain, 1 headache, 1 weight loss	1 cavitations, 2 nodules	2 pulmonary	2 CTX, CLR, and OSE, 2 HCQ, 1 LOP/RIT	2 RIF, INH, PYR, ETB	0
Gadelha Farias et al. [20]	Brazil	2	41	1 HIV, 1 hepatitis B	1 fever, 1 cough, 2 respiratory distress, 1 headache, 1 hemoptysis	2 GGO, 1 cavitation	2 pulmonary	2 HCQ, 2 AZT, 2 CTX	2 RIF, INH, PYR, ETB	0
Yousaf et al. [30]	Qatar	6	35.5	1 diabetes mellitus	3 fever, 5 cough, 2 myalgia, 2 headache, 5 weight loss	6 infiltrations, 6 cavitation, 1 pleural effusion	6 pulmonary	6 (HCQ, AZT, CTX)	6 (RIF, INH, PYR, ETB)	NM
Shabrawishi et al. [24]	Saudi Arabia	7	34.8	1 HIV	7 fever, 6 cough, 2 hemoptysis, 4 night sweats, 6 anorexia, 6 weight loss	1 GGO, 7 consolidation, 3 cavitations, 2 nodules, 1 pleural effusion, 1 pneumothorax	6 pulmonary, 1 both	4 (AZT, CTX), 1 HCQ, 1 (RIB, INH, LOP/RIT)	7 RIF, INH, PYR, ETB	1 death
Khayat et al. [25]	Saudi Arabia	1	40	None	Fever, cough, body aches, chest pain, anorexia	Consolidation	Pulmonary	NM	RIF, INH, PYR, ETB	0
Baskara et al. [31]	Indonesia	1	42	Diabetes	Dizziness, headache, cough, abdominal pain, night sweats	GGO	Pulmonary	AZT, CTX, CTZ, HCQ, OSE	RIF, INH, PYR, ETB	0
Vilbrun et al. [32]	Haiti	1	26	MDR-TB	Fever, cough, dyspnea, weight loss	Cavitation Consolidation	Pulmonary	NM	BDQ, LEV, LNZ, CFZ PYR	0
Luciani et al. [6]	Italy	1	32	BCG vaccination	Fever, myalgia	Consolidation pleural effusion		LOP/RIT, HCQ, LNZ, CLR, TZP	RIF, INH, PYR, ETB	0
Musso et al. [7]	Italy	1	45	Immunosuppression, alcoholism	Fever, cough, fatigue, weight loss, respiratory failure.	GGO, cavitation, hydropneumothorax, atelectasis	Pulmonary	HCQ, corticosteroids	RIF, INH, ETB, PYR, AMK, MOX	Death
Lovino et al. [8]	Italy	1	45	None	Fever, productive cough, hemoptysis, myalgia	GGO, excavated consolidation	Pulmonary	NM	NM	0
Rivas et al. [33]	Panama	2	41	2 HIV	2 fever, 1 cough, 2 dyspnea, 1 weight loss	2 infiltrations	Pulmonary	2 AZT, 2 HCQ, 1 CTX, 1 LEV, 1 antiviral therapy	2 RIF, INH, PYR, ETB	0
Pozdnyakov et al. [34]	Canada	1	64	Diabetes, hypertension, and dyslipidemia	Dyspnea, respiratory distress syndrome	GGO, interstitial syndrome	Pulmonary	CTX	RIF, INH, PYR, ETB	1 death
Faqihi et al. [26]	Saudi Arabia	1	60	Diabetes, hypertension	Fever, cough, chest pain, respiratory distress,	GGO	Pulmonary	LOP/RIT, RIB, DEX	RIF, INH, PYR, ETB	0
Starshinova et al. [35]	Russia	1	59	Heart disease, COPD, emphysema, tuberculosis	Fever, rhinitis, cough, shortness of breath	GGO, infiltration, consolidation, pneumothorax, emphysema	Pulmonary	CTX, LEV, AZT	RIF, INH, PYR, ETB	0
Orozco et al. [27]	Mexico	1	51	Diabetes	Anosmia, dysgeusia, cough, dyspnea	GGO cavitation	Pulmonary	Oxygenotherapy	RIF, INH, PYR, ETB	0
Marwah et al. [13]	India	1	34	MDR tuberculosis, chronic hepatitis B	Productive cough, dyspnea, weight loss, fever, respiratory failure	Cavitation nodules	Pulmonary	Corticosteroids, oxygenotherapy	RIF, INH, ETB, PYR, BDQ	0
PatiL and Gondhali [14]	India	1	75	Ex-smoker	Fever, productive cough, dyspnea, anorexia, weight loss, hemoptysis.	GGO, cavitation infiltrates	Pulmonary	Remdesivir, corticosteroids, anticoagulation	RIF, INH, ETB, PYR	0
Yadav and Rawal [15]	India	1	43	None	Fever productive cough, chest pain, dyspnea, night sweats, respiratory distress.	Bilateral consolidation	Pulmonary	NM	RIF, INH, ETB, PYR	0
Yadav et al. [36]	India	1	26	None	Fever productive cough, chest pain, dyspnea, hemoptysis, weight loss, anorexia.	Consolidation	Pulmonary	NM	INH, ETB, PYR, kanamycin, MOX, CFZ, ethionamide	0
Jacob et al. [37]	India	1	20	Multiple sclerosis	Fever productive cough, weight loss	Opacity	Pulmonary	Corticosteroids, oxygenotherapy, antibiotics	RIF, INH, ETB, PYR	0
Ata et al. [38]	India	1	28	Glioma	Dizziness, headache, vomiting	GGO nodules	Pulmonary with CNS involvement	HCQ, AZT	RIF, INH, PYR, ETB, pyridoxine	0
Goel Sharma et al. [39]	India	1	53	Diabetes chronic kidney disease	Fever, cough, dyspnea, hemoptysis, respiratory distress	GGO, consolidation, fibrosis	Pulmonary	Ampicillin, AZT, HCQ	RIF, INH, PYR, ETB	0
Singh et al. [40]	India	1	76	Hypertension	Fever, respiratory distress, cough, anorexia, weight loss	GGO Consolidation, pleural effusion	Pulmonary	AZT, HCQ, corticosteroids	RIF, INH, PYR, ETB	0
Sahara and Yokota [41]	Japan	1	59	None	Fever, productive cough, hemoptysis, anorexia, taste and smell disorders	GGO, tree-in-bud pattern	Pulmonary	NM	RIF, INH, ETB, PYR	0
Ortiz-Martínez et al. [42]	Colombia	1	34	HIV drug use, anxiety disorder	Fever, dyspnea, headache, cachexia, respiratory distress	GGO, miliary, pleural effusion	Pulmonary	SAM, DOX, corticosteroids, oxygenotherapy	NM	Death
Aissaoui et al. [43]	French Guiana	1	30	NM	Fever, cough, dyspnea, genera status alteration, weight loss, night sweats	Consolidation, tree-in-bud pattern	Pulmonary	CTX, DOX	RIF, INH, ETB, PYR	0
Bouaré et al. [44]	Morocco	1	32	HIV	Fever cough, headache, myalgia.	Miliary	Pulmonary	HCQ, AZT	RIF, INH, ETB, PYR	0
Cutler et al. [45]	USA	1	61	Parkinson	Fever, cough, hemoptysis	Pleural effusion, atelectasis	Pulmonary	HCQ, oxygenotherapy	RIF, INH, ETB, PYR	0
Freij et al. [46]	USA	1	5	Group A streptococcal pharyngitis	Fever, headache	Clear	Extrapulmonary	AMX, HCQ, AZT, corticosteroids, remdesivir	None	Death
Butt et al. [47]	UK	1	42	None	Dyspnea fever, dry cough, fatigue	GGO, Pleural effusion, Consolidation	Pulmonary	DEX, remdesivir	RIF, INH, ETB, PYR	0
Çinar et al. [23]	Turkey	1	55	Myelodysplastic disease, kidney disease, *Klebsiella pneumoniae* infection	Fever, cough, dyspnea	GGO	Disseminated	Plasma therapy, FAV, TCZ, meropenem	RIF, INH, PYR, ETB	0
Tolossa et al. [48]	Ethiopia	1	55	HIV	Fever productive cough, headache, chest pain, dyspnea, night sweats, weight loss, anorexia, fatigue, hemoptysis	Patchy opacities	Pulmonary	Cefepime, corticosteroids	RIF, INH, PYR, ETB	0
Bongomin et al. [49]	Uganda	1	37	HIV, cryptococcal meningitis	Fever productive cough, dyspnea, night sweats, weight loss, anosmia, headache, myalgia, episodes of loss of consciousness.	GGO, miliary	Disseminated	TZP, corticosteroids, oxygenation	None	Death
Wong et al. [11]	Singapore	1	47	None	Fever, chest pain, productive cough, dyspnea.	Opacities cardiomegaly	Extrapulmonary (pericardiac)	Remdesivir, oxygenotherapy	RIF, INH, PYR, ETB	0
Kozinska and Augustynowicz-Kopeć [50]	Poland	2	67.5	1 smoking, 1 HIV, 1 atrial fibrillation,1 renal insufficiency, 2 treated pulmonary TB, treated urogenital TB	1 (cough, fever, dyspnea, sore throat.), 1 no data	1 (atelectasis pleural effusion), 1 no data	1 pulmonary, 1 disseminated	No data	1 (RIF, INH, PYR, ETB), 1 no data	1 death
Gerstein et al. [51]	USA	1	49	Alcoholism cirrhosis	Fever, dry cough, orthopnea, abdominal pain	Pleural effusion, opacities	Disseminated	HCQ, plasma infusion	RIF, INH, ETB, LEV	0
Mulale et al. [52]	Botswana	1	3 months	None	Fever, cough, respiratory distress, failure to thrive	Infiltrates, opacities, consolidations	Disseminated	Ampicillin, gentamicin, oxygenotherapy	RIF, INH, PYR, ETB	Death
Essajee et al. [53]	South Africa	1	2 y 7 months	Cerebral venous thrombosis	Lethargy, hemiplegia, respiratory distress	Miliary	Disseminated	Corticosteroids, oxygenotherapy, antibiotics	RIF, INH, PYR, ethionamide, corticosteroids	0
Brandi et al. [9]	Italy	1	78	Bladder cancer, BCG intravesical instillation, diabetes, COPD, abdominal aortic aneurysm	Fever, cough, dysuria, dyspnea, respiratory distress	GGO, miliary	Disseminated	NM	RIF, INH, ETB	Death
Osejo-Betancourt et al. [54]	Colombia	1	71	Smoking	Fever, dry cough, dyspnea, malaise, anosmia, dysgeusia	Bilateral alveolar opacities, cavitation, consolidation, nodules	Pulmonary	DEX, SAM, oxygenotherapy	RIF, INH, PYR, ETB	0
Pinheiro et al. [21]	Brazil	1	68	Diabetes, hypertension, chronic liver disease	Fever dyspnea, cough,	GGO, opacities, consolidations	Pulmonary	NM	NM	NM
Orozco et al. [27]	Mexico	1	51	Diabetes	Anosmia, dysgeusia, nocturnal diaphoresis, cough, respiratory distress.	GGO, cavitation, nodules	Pulmonary	Oxygenotherapy	RIF, INH, PYR, ETB	0
Gbenga et al. [55]	Nigeria	2	31.5	None	2 fever, 2 cough, 2 weight loss, 1 respiratory distress, 1 sore throat.	1 reticulonodular infiltrates, 1 opacity	Pulmonary	2 AZT, 2 LOP/RIT, 2 (Vit C, zinc sulfate, prednisone)	2 RIF, 2 INH,2 PYR, 2 ETB	0

## Data Availability

The data supporting this case report are from previously reported studies and datasets, which have been cited. The patient's personal data are available from the corresponding author upon request and cannot be disclosed publicly due to the patient privacy policy of our institution.

## Abbreviations

NM: Not mentioned
MDR: Multidrug resistant
COPD: Chronic obstructive pulmonary disease
GGO: Ground-glass opacities
HCQ: Hydroxychloroquine
AZT: Azithromycin
FAV: Favipiravir
LOP/RIT: Lopinavir/ritonavir
MOX: Moxifloxacin
LNZ: Linezolid
UMF HCL: Umifenovir hydrochloride
IFN-*α*: Interferon-*α*
TZP: Piperacillin-tazobactam
CTX: Ceftriaxone
CLR: Clarithromycin
OSE: Oseltamivir
CTZ: Ceftazidime
SAM: Ampicillin-sulbactam
DOX: Doxycycline
DEX: Dexamethasone
TCZ: Tocilizumab
RIB: Ribavirin
RIF: Rifampicin
INH: Isoniazid
PYR: Pyrazinamide
ETB: Ethambutol
STR: Streptomycin
AMK: Amikacin
LEV: Levofloxacin
CS: Cycloserine
BDQ: Bedaquiline
CFZ: Clofazimine.
